# Expression of Legumain Correlates with Prognosis and Metastasis in Gastric Carcinoma

**DOI:** 10.1371/journal.pone.0073090

**Published:** 2013-09-02

**Authors:** Pengtao Guo, Zhi Zhu, Zhe Sun, Zhenning Wang, Xinyu Zheng, Huimian Xu

**Affiliations:** 1 Department of Surgical Oncology, Department of General Surgery, First Affiliated Hospital, China Medical University, Shenyang, China; 2 Lab 1, Cancer Institute, China Medical University, Shenyang, China; Sapporo Medical University, Japan

## Abstract

**Objective:**

Legumain, a novel asparaginyl endopeptidase, has been observed to be highly expressed in several types of tumors, which may play a vital role in carcinogenesis. However, there is no study investigating the relationship among Legumain expression, clinicopathologic, biological variables and patient prognosis in gastric carcinoma.

**Methods:**

In this study, a tissue microarray (TMA) containing 282 samples of primary gastric cancer was assessed for Legumain expression by immunohistochemistry. The TMA included 98 lymph node metastasis samples. The protein expression levels of Legumain were evaluated by Western blot analysis.

**Results:**

Cytoplasmic immunoreactivity of Legumain was over-expressed in gastric cancer compared with paired normal gastric mucosa. Increased Legumain levels were significantly correlated with clinical stage, presence of distant metastasis. Legumain was significantly over-expressed in primary gastric cancer with metastasis than without metastasis. Patients with Legumain-positive localized tumors had lower 5-year overall survival (OS) than those with Legumain-negative tumors. Multivariate survival analysis showed that Legumain was an independent prognostic marker for OS (HR 1.459, 95% CI 1.251–1.703, P = 0.007).

**Conclusions:**

Legumain expression could serve as a prognostic biomarker in patients at risk of developing metastasis or recurrence with gastric carcinoma.

## Introduction

Legumain is a cysteine endopeptidase of the asparaginyl endopeptidase family, displaying high specificity for hydrolysis of asparaginyl bonds. This lysosomal protease belongs to the peptidase family C13. Legumain was found to be highly expressed in several types of tumors, such as colon, prostate and breast cancers [1], [2], [3]. Its expression was observed to be up-regulated during tumor development in vivo, suggesting an environmental response, and seems to be a stress-responsive gene, being markedly elevated in cells subjected to environmental stress [4]. Legumain is expressed both intracellular and on the cell surface by tumor cells and tumor associated endothelial cells, where it is colocalized with integrins [5]. It is found especially in membrane-associated vesicles concentrated at the invadopodia of tumor cells. Notably, cell surface proteases are often associated with invasive and metastatic tumor cells [6]. Cells that highly express Legumain exhibit enhanced migratory and invasive properties [7]. These properties may be mediated by increased extracellular matrix degradation, resulting from activation of zymogens such as progelatinase A. Legumain activates the gelatinase A zymogen, an important mediator of extracellular matrix degradation, and thus may be important for tumor cells to adapt a more invasive and metastatic phenotype [8]. Animal tumor models generated with cells over expressing Legumain showed an in vivo behavior that is vigorous with more increased invasive growth and metastasis [9]. This phenotype is proposed to result from the proteolytic function of Legumain that result in activation of other protease zymogens. Some proteases are linked to other properties of tumors such as angiogenesis and growth signaling and may be activated by Legumain. Protease cascades are characteristic of many biological pathways such as the coagulation, apoptosis, and complement cascades [10]. Legumain may represent a target for inhibition of tumor growth and metastasis based on its enhancement of tumor growth and its unique restricted specificity.

These studies show that increased expression of Legumain may play a role in promoting tumor progression and suggest that tumors expressing high levels of Legumain should be expected to display a more aggressive behavior and have a poor prognosis. The aim of the present study was to test this hypothesis by examining expression of Legumain in primary gastric cancers, normal mucosa, and lymph nodes metastasis, and to determine the relationships between Legumain expression and various clinicopathologic and biological variables.

## Materials and Methods

### Patients and Study Design

A total of 282 patients who had surgery for gastric cancer between January 2006 and December 2009 at the First affiliated hospital of China Medical University was selected for this study. The group was composed of 205 men and 77 women with a mean age of 65±15 (range, 22–95) years. 188 of these patients had lymph node metastasis. The tissue microarrays (TMAs) were made from paired tumor and non-tumor tissue from each patient and included the LNM samples for comparison with the primary tumor tissue. Immunohistochemistry and Western blot was performed to evaluate Legumain expression in cell lines and all patients. Correlations between Legumain expression levels and survival were analyzed.

### Ethics Statement

Ethical approval for this research was obtained from the Research Ethics Committee of China Medical University, China. All patients providing tumor tissue as well as normal gastric tissue samples signed a consent form prior to surgical removal of the gastric carcinoma to allow for this research to be undertaken.

### Tissue Samples and Cell Lines

All patient-derived specimens were collected and archived under protocols approved by the Institutional Review Boards of the First affiliated Hospital China Medical University. The diagnoses were confirmed by at least two pathologists and staging was based on pathological findings according to the American Joint Committee on Cancer (AJCC) guidelines [11]. The median duration of follow-up was 51 (range, 5–78) months.

Normal gastric cell line GES-1 and gastric cancer cell lines MKN28 (well differentiated), AGS, SGC-7901 (moderately differentiated), MGC-803 (poorly differentiated) were all purchased from the cell bank of Chinese Academy of Sciences. GES-1 was maintained in RPMI 1640 (Hyclone, Logan city, USA) supplemented with 20% fetal bovine serum (FBS). Five cancer cell lines were maintained in RPMI 1640 (Hyclone, Logan city, USA) supplemented with 10% FBS. All the cell lines were in a 5% CO2 humidified atmosphere at 37°C.

### Follow-Up after Surgery

The 282 patients who underwent gastroctomy were subjected to close clinical observation, including chest/abdominal/pelvic computed tomographic (CT) imaging, CEA level, and blood testing at 2- to 3-month intervals and a yearly gastroscopy. Follow-up was in accord with National Comprehensive Cancer Network (NCCN) Practice Guidelines in gastric cancer. Overall survival rates were defined as the interval from the initial surgery to clinically or radiologically proven recurrence/metastasis and death, respectively. The end date of the follow-up study for conducting the analysis was June 29, 2012.

### TMA Construction and Immunohistochemistry

Hematoxylin and eosin (H&E)-stained slides were screened for optimal tumor tissue and noncancerous tissue adjacent to tumor (at least 2 cm from the tumor) and TMA slides were constructed with a tissue manual arraying instrument. Two cores were collected from each formalin-fixed, paraffin-embedded (FFPE) gastric cancer tissue sample and from each normal gastric mucosa sample using a 1.0-mm diameter punch instrument. At least one LNM core was collected in the same manner. Samples from the same patient were spotted next to each other to ensure similar reaction conditions for the normal and tumor tissue of that patient. Immunohistochemical Analysis was performed on FFPE samples as described previously using an Envision kit (Dako Cytomation, Glostrup, Denmark). Pressure cooker mediated antigen retrieval was performed in citrate buffer (pH 6.0) for 7 min. Sections were incubated with 1∶200 dilution of anti-Legumain (Santa Cruz, California, US) overnight at 4°C, and then incubated with goat anti-mouse or anti-rabbit Envision System Plus-HRP (Dako Cytomation) for 30 min at room temperature. After rinsing three times in PBS for 10 min each, the sections were incubated with DAB for 1 min, counterstained with Mayer hematoxylin, dehydrated, and mounted.

### Evaluation of Immunohistochemical Staining

Immunoreactivity was evaluated independently by two researchers who were blinded to patient outcome. The evaluation was based on the staining intensity and extent of staining. Staining intensity for Legumain was scored as 0 (negative), 1 (weak), 2 (moderate), and 3 (strong). Staining extent was scored as 0 (0%), 1 (1–25%), 2 (26–50%), 3 (51–75%), and 4 (76–100%), depending on the percentage of positive-stained cells. The sum of the staining intensity and the staining extent scores was used as the final staining score. The specimens were divided into three groups according to their overall scores: 0–1, negative, 2–4, weak positive, and 5–7, strong positive. In the event of a discrepancy in scoring, the slides were reexamined by both pathologists under a multihead microscope.

### Western Blot and Realtime PCR

Cells were washed twice with cold PBS and lysed on ice in RIPA buffer with protease inhibitors and quantified by BCA method. 50 mg Protein lysates were resolved on 6% SDS polyacrylamide gel, electrotransferred to polyvinylidene fluoride membranes (Millipore, Bedford, MA) and blocked in 5% nonfat dry milk in Tris-buffered saline (pH = 7.5). Membranes were immunoblotted overnight at 4°C with anti-Legumain polyclonal antibodies as IHC described above, respectively, then followed by their respective secondary antibodies. Signals were detected by enhanced chemiluminescence (Pierce, Rockford, IL).

PCR amplifications for quantification of Legumain and β-actin mRNA in cells were done in a LightCycler system (Roche Applied Science) using the LightCycler FastStart DNA Master SYBR Green I kit (Roche Diagnostics). In brief, a master mixture was prepared on ice, containing 1 ml of complementary DNA, 2 ml of LC DNA Master SYBR Green I mix, 50 ng of primers, and 4 mM MgCl2. The amplification conditions for 40 cycles consisted of denaturation at 95°C for 10 s, annealing at 65°C for 10 s, and extension at 72°C for 10 s. The products were then subjected to a temperature gradient from 68 to 95°C at 0.1°C/s, with continuous fluorescence monitoring to produce melting curves of the products. The sequences of the primers used are as follows:

Legumain forward primer, 5′-GATGAACCACCTGCCGGATAA-3′, Legumain reverse primer, 5′-CATCATAGTAACAGGCGTAGGACGA-3′, Data were analyzed according to the comparative Ct method and were normalized to β-actin expression in each sample.

### Statistical Analysis

The χ2 test or Fisher’s exact test for proportion was used, as appropriate, to analyze the relationship between Legumain expression and clinicopathological variables. The survival rates were calculated by the Kaplan–Meier method and the differences between the survival curves were examined by the log-rank test. Univariate Cox proportional hazards regressions were applied to estimate the individual hazard ratio (HR). The significant variables in the univariate analyses (P<0.05) were then put into the multivariate analysis. The HR with 95% confidence interval (CI) was measured to estimate the hazard risk of individual factors. P<0.05 was considered to be statistically significant. Analyses were performed using the SPSS statistical software program version 19.0 (SPSS Inc., Chicago, IL).

## Results

### Legumain Expression in Human Gastric Cancer Tissue

**Figure 1:**
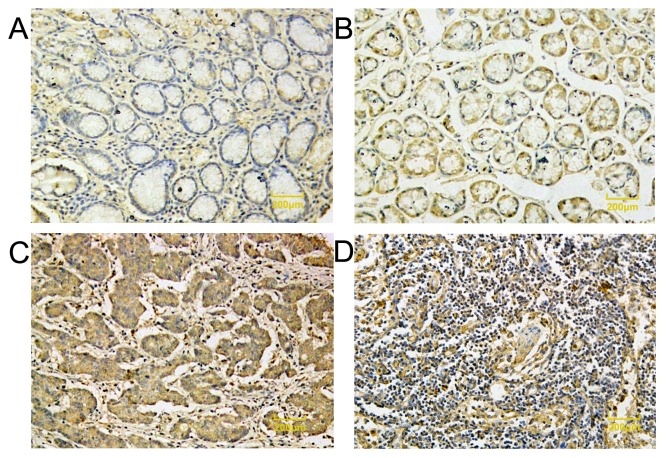
Analysis of Legumain protein expression by immunohistochemistry in normal gastric mucosa, gastric cancer and lymph node metastasis. (a) (b) Legumain expression exhibited negative or diffuse weakly positive staining in the cytoplasm in distant normal mucosa. (c) Legumain exhibited strong vesicular positivity in the cytoplasm in primary gastric cancer compared with those of adjacent normal mucosa. Vesicles were scattered in the cytoplasm. (d). Legumain also overexpressed in metastasis lymph nodes. (magnification ×100).

Legumain both expressed in noncancerous mucosa, primary gastric cancer tissue, and metastasis lymph nodes. Legumain expression exhibited negative or diffuse weakly positive staining in the cytoplasm in distant normal mucosa and exhibited strong vesicular positivity in the cytoplasm in primary gastric cancer. Vesicles were scattered in the cytoplasm. Legumain overexpressed in metastasis lymph nodes. Figure 1 represents the immunostaining profiles of Legumain in gastric cancer. Only 45 of the 181 normal gastric mucosa samples (24.5%) showed weak Legumain immunoreactivity, and the rest were Legumain negative. Among the 282 samples of gastric cancer, 86 (29.8%) were weakly positive, 128 (45%) were strongly positive, and 68 (25.2%) were undetectable. These studies indicated that Legumain expression was significantly upregulated in cancerous tissue compared with the corresponding noncancerous normal mucosa (P<0.001).

**Figure 2:**
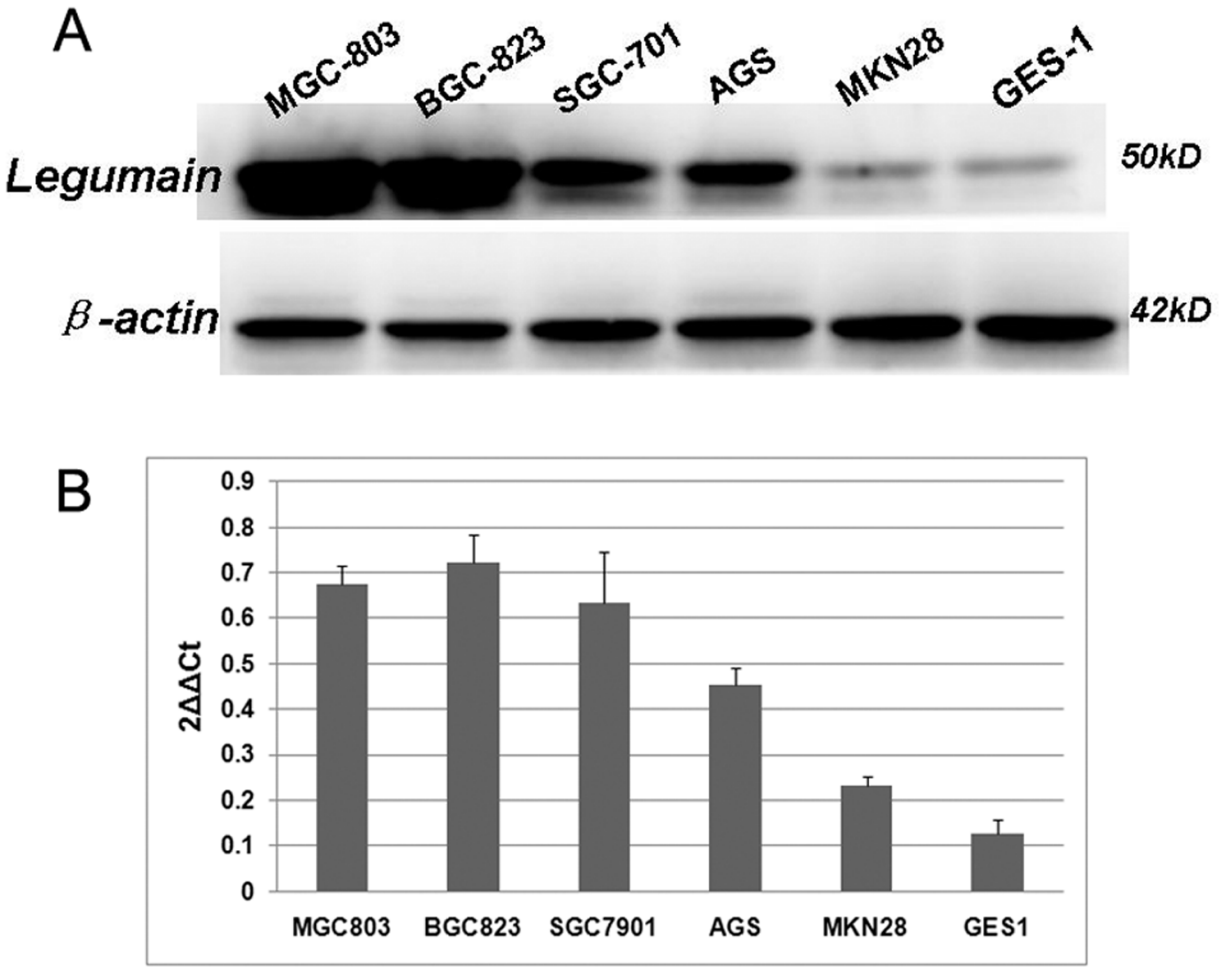
Legumain expression at the mRNA and protein levels. (a) Western blotting analysis of Legumain in normal gastric cell lines (GES-1) and gastric cancer cell lines (MKN28, AGS, SGC-7901, MGC-803, BGC-823). BGC-823, MGC-803 cell (poorly differentiated) and AGS, SGC-7901 (moderately differentiated) showed lower expression of Legumain in comparison with GES-1 (normal gastric cell lines) and MKN28 (well differentiated), (b) β-actin is the loading control. Legumain expression was confirmed in all the cell lines by realtime PCR.

Western blotting analysis of Legumain in normal gastric cell lines (GES-1) and gastric cancer cell lines (MKN28, AGS, SGC-7901, MGC-803, BGC-823). BGC-823, MGC-803 cell (poorly differentiated) and AGS, SGC-7901 (moderately differentiated) showed lower expression of Legumain in comparison with GES-1 (normal gastric cell lines) and MKN28 (well differentiated) (Fig. 2), confirming the results of the immunostaining and realtime PCR study.

Lymph node metastasis is a key step in the metastatic cascade in gastric cancer, we evaluated Legumain expression in matched primary gastric cancer and lymph node metastatic sites. Of the 98 cases of metastasis lymph nodes analyzed, 35 samples (35.7%) showed Legumain over-expression (Table 1) compared with the paired primary gastric cancer, of which 45% showed Legumain over-expression (P<0.001). Legumain protein expression was elevated in metastasis lymph nodes samples compared with primary tumor samples in 35 of 98 pairs. In 42 pairs, there was no difference in Legumain expression levels, and in 21 cases, Legumain expression levels were decreased in the metastasis.

**Table 1:** Expression of Legumain in normal colonic mucosa, cancerous tissue and lymph node metastasis.

Tissue Sample	n	Legumain			P value
		Negative(%)	Weak(%)	Strong(%)	
Adjacent Normal Mucosa	181	136(75.1)	45(24.9)	0(0)	
Primary Cancer Tissue†	282	68(25.2)	86(29.8)	128(45)	<0.001
Lymphnode‡	98	21(21.4)	42(42.8)	35(35.7)	<0.001

P values are based on Fisher’s exact test. Multiple comparisons between groups were determined with p = 0.01 adjustment, due to multiple hypotheses.

† Significant difference in the expression of Legumain between noncancerous mucosa and the gastric cancerous tissue sample.

‡ Significant difference in the expression of Legumain between noncancerous mucosa and the Lymphnode tissue sample.

### Immunohistochemical Findings

Table 2 shows the clinicopathological significance of Legumain expression. Over-expression of Legumain was significantly associated with lymph node metastasis (P<0.001), peritoneal metastasis (P = 0.002), hepatic metastasis (P = 0.014) and histologic type (P = 0.095), the T stage (P = 0.003). Among the Legumain-positive samples, 164 of 188 (87.2%) were positive for lymph node metastasis compared with the Legumain negative group, of which 39 of 41 samples (94.8%) showed peritoneal metastasis, 34 of 37 samples (91.6%) showed hepatic metastasis, suggesting a potential role for Legumain in the promotion of cancer cell proliferation and growth.

**Table 2:** Association between Legumain expression and clinicopathologic factors in gastric cancers.

Variables	Cases	Legumain		P value
		−	+	
		68	214	
Gender				0.166
Female	77	23	54	
male	205	45	160	
Age(years)				0.809
≤65	182	38	144	
>65	100	30	70	
Location				0.12
Lower	217	40	177	
Middle	21	11	10	
Upper	39	15	24	
Entire	5	2	3	
Macroscopic Type				0.036
Early stage	31	8	23	
Borrmann I	4	3	1	
Borrmann II	20	8	12	
Borrmann III	199	43	156	
Borrmann IV	28	6	22	
Histologic type				0.095
Differentiated	124	35	89	
Undifferentiated	158	33	125	
Lauren grade				0.652
Intestinal	137	22	115	
Diffuse	145	46	99	
T Stage				0.003*
T1	28	10	18	
T2	50	25	25	
T3	184	26	158	
T4	20	7	13	
N Stage				<0.001*
N0	96	51	45	
N1	45	3	42	
N2	53	7	46	
N3	88	7	81	
Peritoneal metastasis				0.002*
Absent	241	66	175	
Present	41	2	39	
Hepatic metastasis				0.014*
Absent	245	65	178	
Present	37	3	34	

P values are based on X^2^ test,

* Significant difference were determined with p<0.05.

### Survival Analysis

The 5-year OS rate of the 282 patients with primary gastric cancer was 52.4% (148/282), with 55 deaths observed during the follow-up period. Importantly, we found that Legumain-positive and Legumain-negative patients exhibited a significant difference in the occurrence of metastasis after curative gastrectomy. More patients with Legumain-positive tumors (125/224 patients (55.8%), mean follow-up, 63 (range, 57–69) months) subsequently developed metastasis than those with Legumain negative tumors (22/76 patients (28.9%), mean follow-up, 85 (range, 83–87) months, P<0.01).

**Figure 3:**
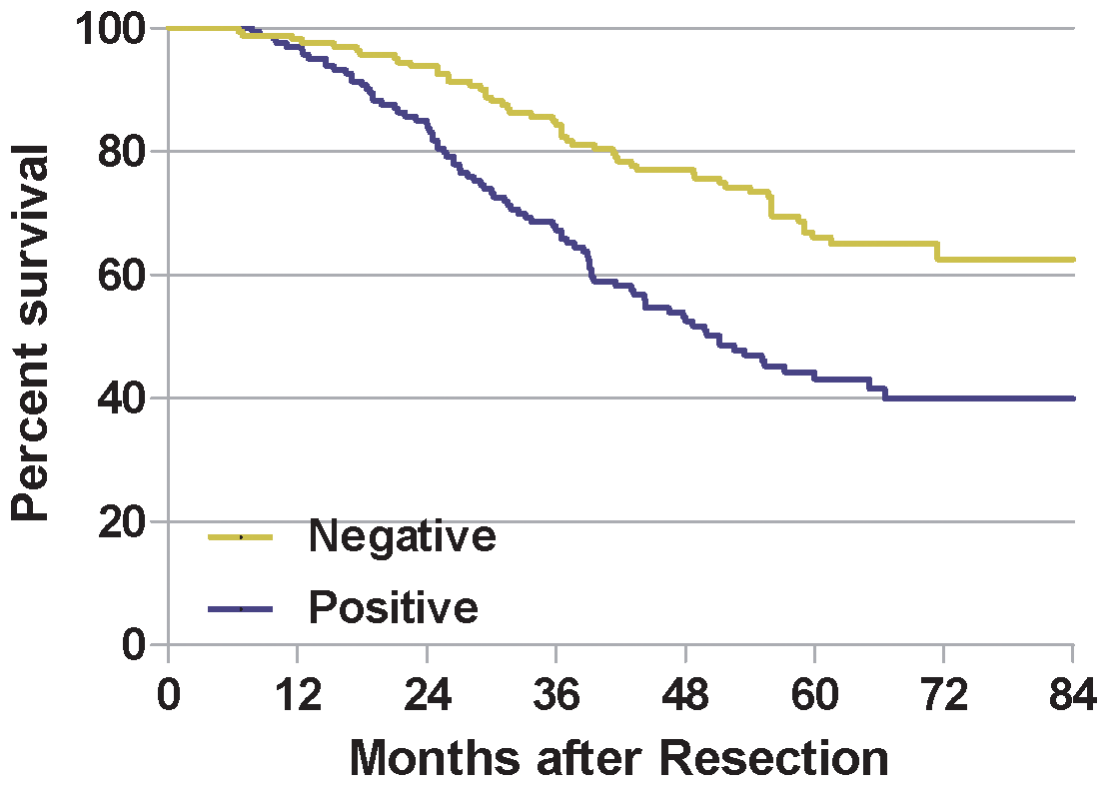
Correlation of Legumain expression with survival curves of patients with gastric cancer. Patients with Legumain-positive expression showed significantly better disease-free survival (a) and overall survival (b) than those with Legumain-negative expression (P<0.001, log-rank test).

On univariate analysis, patients whose localized gastric tumors were Legumain-positive had a significantly lower 5-year OS than those with Legumain-negative tumors (51.1 vs.78.2%, HR 1.711, 95% CI 1.486–1.970) (Fig. 3, Table 3). In addition, distant metastasis and T stage were associated with OS.

**Table 3:** Univariate and multivariate analysis of overall survival after surgery.

Variables	Univariate analysis		Multivariate analysis	
	HR (95% CI)	P value	HR (95% CI)	P value
Legumain	1.711 (1.486–1.970)	<0.001*	1.459 (1.251–1.703)	0.007*
Histologic grade	1.285 (0.922–1.790)	0.139	1.066 (0.746–1.522)	0.726
T Stage	1.957 (1.511–2.534)	<0.001*	1.559 (1.109–2.193)	0.011
N Stage	1.826 (1.274–2.615)	<0.001*	1.573 (1.073–2.306)	0.02
Peritoneal metastasis	2.057 (1.530–2.765)	<0.001*	1.799 (1.315–2.462)	<0.001*
Hepatic metastasis	1.406 (1.011–1.956)	0.043	1.315 (0.935–1.850)	0.116

HR: hazard radio, CI: confidence interval,

* Significant difference.

Multivariate analysis was performed using the Cox proportional hazards model for all of the significant variables in the univariate analysis. The results from the multivariate analysis showed that Legumain expression (P = 0.007), peritoneal metastasis (P<0.001) were significant independent prognostic factors for OS (Table 3).

## Discussion

Legumain is an identified lysosomal protease, a novel member of the C13 family of cysteine proteases, which are well conserved, present in plants, invertebrate parasites, as well as in mammals, and have a highly restricted specificity requiring an asparagine of substrates [12]. Proteases have been implicated in many aspects of tumor cell biology [13]. Thus, a protease that is highly expressed by tumor cells or tumor vascular endothelial cells might contribute to tumor cell progression through processing of signaling molecules and their receptors, thereby influencing cellular responses [14]. Such effects might also result in diminished apoptosis, thereby enhancing tumor growth. Legumain has been detected in several types of human cancer including breast carcinomas, colon carcinomas, and central nerve system neoplasms [1], [15], here, we present evidence that Legumain is over expressed in the gastric cancer, whereas Legumain was weakly expressed or not observed in the distant normal tissues of tumor derivation. We demonstrate that over expression of Legumain may be associated with enhanced tissue invasion and metastasis. Its unique functional properties and high-level expression in many human tumors may lend itself as an apparent regulator of cellular behavior in migration and tissue invasion. Our results suggest that Legumain might be a novel molecular marker for gastric cancer.

We examined the expression of Legumain in nodal metastasis as in the primary gastric cancer specimens. This strongly suggests that Legumain may play a critical role in the development of metastasis and invasive behavior. Cells that highly express Legumain exhibited enhanced migratory and invasive properties. A correlation between tumor invasion and metastasis with the presence of cysteine endopeptidases has been observed [16]. Hydrolysis of asparaginyl bonds is prominent in the posttranslational processing of cathepsin. Legumain might activate local cysteine protease zymogens to their active two-chain protease forms. Protease zymogens are dependent on limited proteolytic activation for conversion to the functional state. Protease cascades are characteristic of many biological pathways such as the coagulation, apoptosis, and complement cascades. Similar cascades appear to be involved in tumor invasion and metastasis [17]. Characterization of the later is complicated by the diversity of neoplasms. However, comprehensive profiling of protease expression and function may advance understanding of tumor invasion and metastasis.

We furthermore investigated the relationship of Legumain expression with clinicopathologic and biological variables and found that abundant expression of the Legumain was related or tended to show a positive correlation with clinical stage and grade, increased regional lymph node involvement, and distant metastasis in advanced gastric carcinoma. Additionally, Legumain over expression was highly associated with increased cell migration and malignant transformation leading to lymph node involvement and increased invasiveness. In our study, we showed a significant correlation between elevated Legumain expression and T stage (P = 0.003), lymph node metastasis (P<0.001), peritoneal metastasis (P = 0.002) and hepatic metastasis (P = 0.014). This strong correlation suggests that Legumain can be used as a biomarker to identify subsets of patients with gastric cancer with a more aggressive phenotype. We propose that Legumain over-expression may contribute to increased proliferation and the development of gastric cancers.

Metastasis and local recurrence are the critical determinants of death after gastrectomy in gastric cancer patients. They are considered as associated properties of tumor cells because they use similar processes involving physical attachment of cells to their environment through cell adhesion molecules and activation of extracellular proteases. Increased expression of proteases and downregulation of protease inhibitors is commonly observed in tumors. Notably, cell surface proteases are often associated with invasive and metastatic tumor cells. Some proteases are linked to other properties of tumors such as angiogenesis and growth signaling as perhaps with Legumain. Our data showed Legumain expression as an important predictor for metastasis in gastric cancer. Legumain-positive patients exhibited a more aggressive form of disease those with Legumain negative tumors, with a higher risk of distant metastasis. We also showed that Legumain could serve as a prognostic marker to predict the risk of metastasis and recurrence with a HR of 1.711 for OS. Previous research showed that 60% of Legumain-positive patients with superficial invasive urothelial carcinoma at initial diagnosis went on to develop metastasis, whereas no metastasis were found in Legumain-negative patients [18]. This means that Legumain expression could be used at the time of initial diagnosis to not only to design optimal, individualized treatment, but also to distinguish those patients who would benefit from closer monitoring after surgery.

Some metalloproteinase inhibitors have demonstrated tumor stasis in animal models [19]. Similarly, Legumain may represent a target for inhibition of growth and metastasis based on its up-regulation associated with tumor growth and unique restricted specificity. The high level of Legumain expression by tumor cells coupled with its unusual and highly specific substrate requirement for catalytic function makes it an attractive candidate for prodrug conversion in a therapeutic mode [20], [21].

In summary, it has been shown highly expressed of Legumain has a negative influence on prognosis and a positive correlation with metastasis of gastric carcinoma, possibly through increased extracellular matrix degradation. We propose that Legumain could serve as a biomarker to predict prognosis in patients who undergo curative gastrectomy, and further studies are needed to clarify the role of Legumain in the progression of gastric cancer.
